# Glial cells react to closed head injury in a distinct and spatiotemporally orchestrated manner

**DOI:** 10.1038/s41598-024-52337-4

**Published:** 2024-01-30

**Authors:** Ester Nespoli, Marsela Hakani, Tabea Melissa Hein, Stephanie Nadine May, Karin Danzer, Thomas Wirth, Bernd Baumann, Leda Dimou

**Affiliations:** 1 Molecular and Translational Neuroscience, Department of Neurology, Ulm University, Ulm, Germany; 2 Institute of Physiological Chemistry, Ulm University, Ulm, Germany; 3 Department of Neurology, Ulm University, Ulm, Germany; 4 German Center for Neurodegenerative Diseases (DNZE), Ulm, Germany

**Keywords:** Cell biology, Neuroscience, Medical research, Neurology, Pathogenesis

## Abstract

Traumatic brain injury (TBI) is a leading cause of mortality and disability worldwide. Acute neuroinflammation is a prominent reaction after TBI and is mostly initiated by brain-resident glial cells such as microglia, NG2-glia and astrocytes. The magnitude of this reaction paves the way for long-lasting consequences such as chronic neurological pathologies, for which therapeutic options remain limited. The neuroinflammatory response to TBI is mostly studied with craniotomy-based animal models that are very robust but also rather artificial. Here, we aimed to analyze the reaction of glial cells in a highly translational but variable closed head injury (CHI) model and were able to correlate the severity of the trauma to the degree of glial response. Furthermore, we could show that the different glial cell types react in a temporally and spatially orchestrated manner in terms of morphological changes, proliferation, and cell numbers in the first 15 days after the lesion. Interestingly, NG2-glia, the only proliferating cells in the healthy brain parenchyma, divided at a rate that was correlated with the size of the injury. Our findings describe the previously uncharacterized posttraumatic response of the major brain glial cell types in CHI in order to gain a detailed understanding of the course of neuroinflammatory events; such knowledge may open novel avenues for future therapeutic approaches in TBI.

## Introduction

Traumatic brain injury (TBI), with approximately 50–60 million new cases per year, is a leading cause of mortality and disability across all ages worldwide. Low- and middle-income countries, where trauma prevention measures are often insufficient, pay the highest death toll^1^. Additionally, in industrialized countries, TBI represents a large burden on the healthcare system because survivors require specialized long-term rehabilitation and are highly susceptible to neurological diseases such as dementia, Alzheimer’s disease, and epilepsy, along with depression and other cognitive disturbances^2–4^.

TBI is caused by an external force that can mechanically deform brain tissue. Its severity can be classified as mild, moderate, or severe according to the patient’s consciousness level using the Glasgow Coma Scale, whose score strongly correlates with the outcome of trauma. While mild TBI typically causes only limited acute symptoms and almost no tissue damage, long-term effects have been reported in association with repetitive trauma; however, these long-term effects are still a matter of speculation. In contrast, moderate and especially severe TBI causes widespread tissue damage, skull fractures (hairline cracks), hematoma formation, and, in 30–40% of cases even leads to death^5,6^.

Therapeutic options for TBI remain limited, and recent clinical trials have failed despite promising preclinical results. One of the major problems in the field remains the narrow translational potential of the most commonly used animal models of TBI, as most of them, including the controlled cortical impact (CCI), stab wound injury (SWI), and fluid percussion injury (FPI) models are based on a craniotomy. On the one hand, these models are highly appreciated for their reproducibility; on the other hand, however, they fail to replicate the complex etiology of human brain trauma^7–9^.

Another critical obstacle to the discovery of new therapies for TBI is our limited knowledge regarding the kinetics of the cellular processes that initiate and govern the TBI response and, importantly, determine its outcome. As the brain is an immune-privileged organ, the cellular immune response to TBI is carried out primarily by resident glial cells, such as microglia, astrocytes, and NG2-glia.

Microglia, the resident immune cells of the brain, react rapidly to all forms of brain injury. Within minutes of sensing tissue damage, they modify their morphology and initiate inflammatory processes by releasing cytokines and chemokines, removing cellular debris, and contributing to the remodeling of the extracellular matrix. When the acute trauma reaction is complete, microglial proinflammatory action can persist and cause chronic inflammation as well as long-term neural damage^10,11^.

Under physiological conditions, NG2-glia, also known as oligodendrocyte progenitor cells, are the only proliferating cells in the adult brain parenchyma, outside the neurogenic niches^12^. This unique characteristic allows them to keep their population constant, although they are primarily known for their lifelong ability to differentiate into oligodendrocytes^13,14^. Even though their regenerative capacity and progenitor status are of clear interest for trauma research, NG2-glia are still understudied compared to other glial cells. Their early polarization, migration, and proliferation around the trauma site, as shown in recent reports, have brought NG2-glia to the spotlight as key contributors to wound closure in the brain^12,15,16^.

Astrocytes are the most abundant population of glial cells and are responsible for numerous homeostatic functions in the brain. Their acute reaction to various types of brain insults is evidenced by their expression of biomarkers such as glial fibrillary acidic protein (GFAP), which can be upregulated by subsets of astrocytes in the cerebral gray matter even after mild trauma. Upon more severe trauma, GFAP is released into the serum and can be identified there; this principle underlies the use of GFAP as a biomarker after TBI in human. Reactive astrogliosis as the result of moderate or severe trauma is also characterized by the secretion of inflammatory mediators, changes in morphology, and cell proliferation. The most characteristic contribution of astrocytes to trauma resolution is that they coordinate the formation of a glial scar that segregates injured tissue from healthy tissue, thus limiting the spread of damage^17,18^.

The above-described glial cells can engage in mutual crosstalk and react to an injury to limit tissue damage and re-establish homeostatic functions. To date, studies on the reaction of these cells to injury have mostly focused on a single cell type at a time and predominantly used CCI to model brain trauma. In our study, we addressed the individual and coordinated reaction of microglia, astrocytes, and NG2-glia in a closed head injury (CHI) model, as the strictly timed reaction of each cell type could contribute differently to trauma resolution. Furthermore, we aimed to investigate common trauma parameters that can influence the overall glial cell reaction and could be predictive of the posttraumatic outcome. For this reason, we chose to use a weight-drop CHI mouse model that involves skull exposure but not skull removal and mimics the nature and heterogeneity of human TBI^19–21^. We observed that the overall glial cell reaction is absent when only skull fracture occurs, while the reaction develops with increased severity of the injury when bleeding also takes place. In the latter case, microglia and NG2-glia are the first cell types to respond to TBI through morphological changes, with the changes beginning one day after TBI, while they reach their maximal proliferation rate only at approximately 3 days after TBI. In contrast, astrocytes react through GFAP upregulation and proliferation later, between 3 and 7 days after trauma. We confirmed the previous observation that scar formation is already visible at 7 days after trauma, and we showed that both the total number of NG2-glia and, more interestingly, the number of actively proliferating NG2-glia correlate directly with the dimensions of the insult, suggesting that NG2-glia are a cell type to watch closely in preclinical trauma research.

## Results

### Glial cell reaction to TBI is elevated upon increased severity of the injury

**Figure 1:**
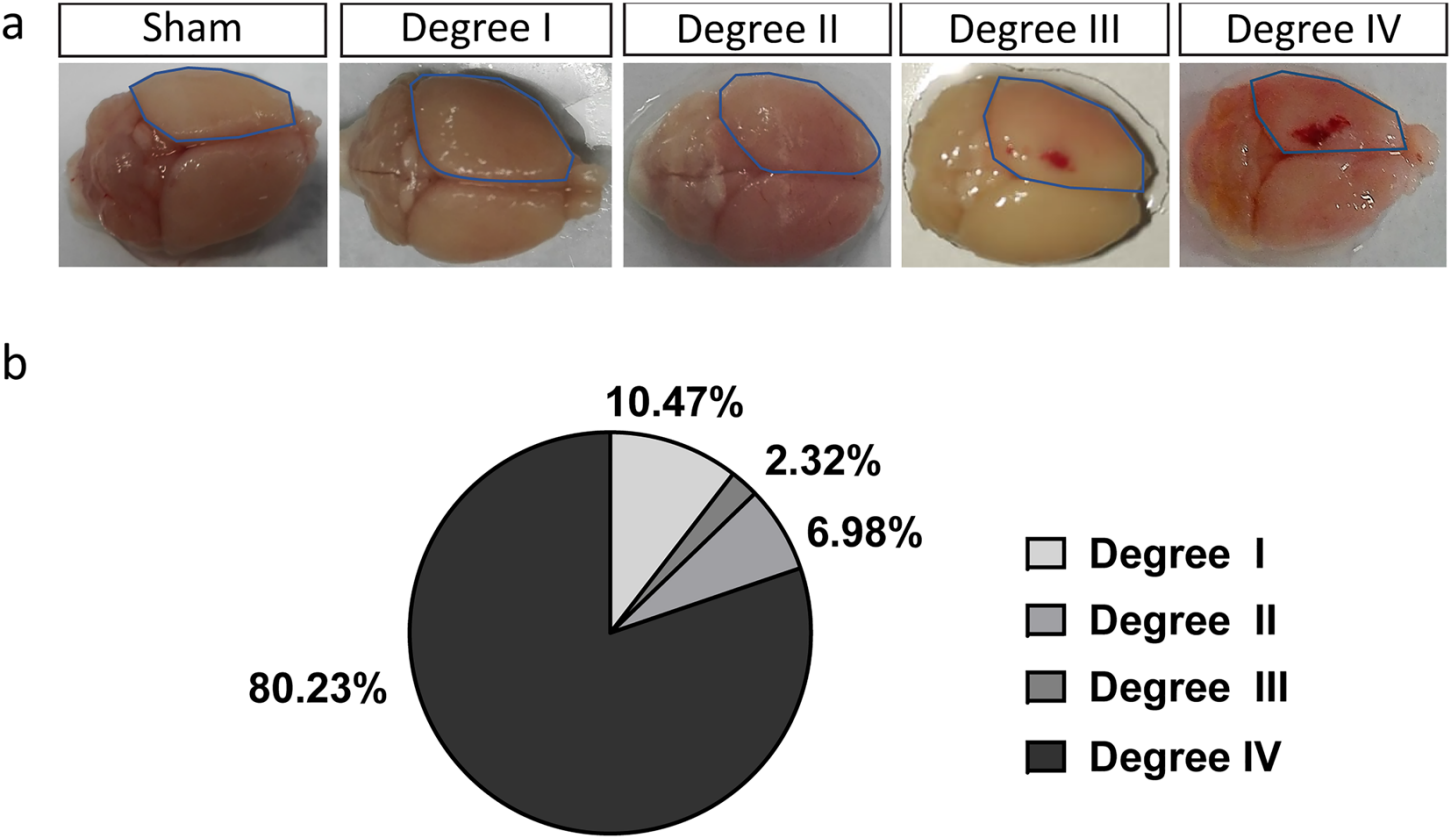
CHI can lead to different degrees of injury severity. (**a**) Three days after CHI, the presence of fracture and bleeding (degree IV), only bleeding (degree III), only fracture (degree II), or neither (degree I) could be determined visually on the ipsilateral side of the injury (dotted blue line). (**b**) Percentages of the different outcomes after CHI.

**Figure 2:**
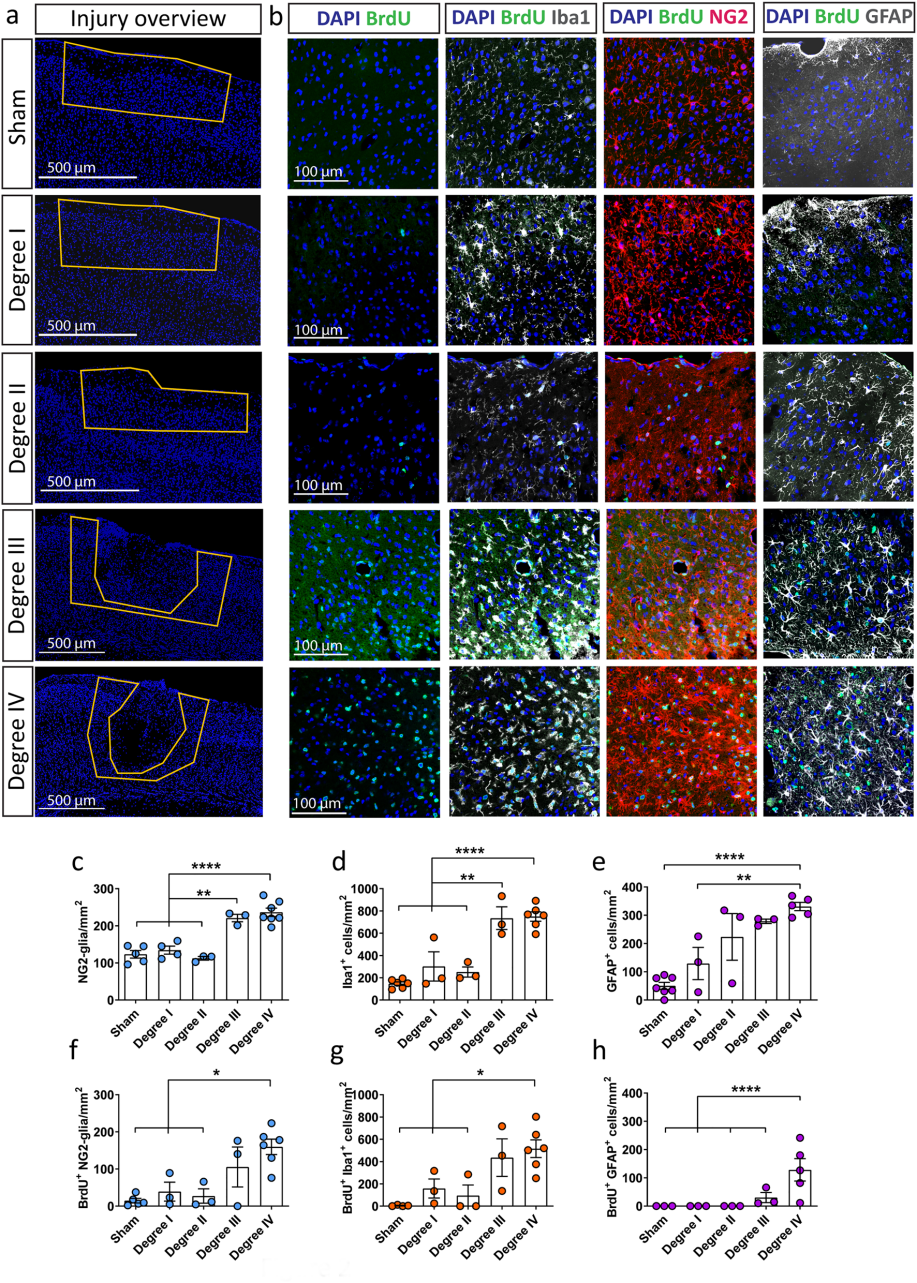
The response of glial cells to CHI is elevated with increased severity of the injury. (**a**) Immunohistochemical analysis which shows the injury overview with the use of the nuclear marker DAPI (blue). The yellow line highlights the area that was chosen for analysis regarding cell imaging and quantification. (**b**) Representative IHC images of higher magnification within the area that was analyzed; BrdU^+^ cells (green) alone or in combination with NG2-glia (red, middle panel), Iba1^+^ cells (microglia, white, left panel), and GFAP^+^ cells (reactive astrocytes, white, right panel) at 3 days after CHI according to the trauma severity. Quantitative analysis of (**c**) NG2-glia, (**d**) Iba1^+^ cells, and (**e**) GFAP^+^ cells in the different degrees of CHI. Quantitative analysis of (**f**) BrdU^+^ NG2-glia, (**g**) BrdU^+^ Iba1^+^ cells, and (**h**) BrdU^+^ GFAP^+^ cells in the different degrees of CHI. Statistical analysis: one-way ANOVA with Tukey's post hoc test; *p < 0.05, **p < 0.01, ****p < 0.0001. Data are presented as the mean ± SEM.

To understand the reaction of glial cells to TBI, we used a weight-drop CHI mouse model. This kind of mild injury resulted in the following outcomes regarding the severity degrees: absence of skull fracture or bleeding (mild trauma degree I), only hairline skull fracture (mild trauma degree II), intracerebral bleeding without skull fracture (mild trauma degree III) and finally the more severe paradigm where hairline skull fracture and intracerebral bleeding (mild trauma degree IV) was observed (Fig. 1a,b). The acute cellular reactions associated with the different outcomes of CHI were studied at 3 days after CHI, a timepoint that has previously been associated with the maximal glial reaction after SWI and CCI^12,22^. In the area directly adjacent to the trauma (yellow area in Supplementary Fig. S1), we immunostained and quantified the glial populations that are mostly involved in TBI repair mechanisms: microglia (Iba1^+^ cells), NG2-glia (NG2^+^ cells), the complete population of astrocytes (S100β^+^ cells) and reactive astrocytes (GFAP^+^ S100β^+^ cells) (Fig. 2; Supplementary Fig. S2). The breakdown of the blood–brain barrier (BBB) in injuries of degree IV was verified with IgG immunohistochemistry (IHC; Supplementary Fig. S3) and was associated with an increased concentration of Iba1^+^ microglia as well as NG2-glia around the trauma site (Fig. 2). While the absolute numbers of the complete population of astrocytes (S100β^+^) was not significantly changed after injury (Supplementary Fig. S2) we could observe an increase in the number of reactive astrocytes as shown by the expression of the GFAP marker (S100β^+^ GFAP^+^ astrocytes; Fig. 2; Supplementary Fig. S2), a protein not expressed in cortical grey matter astrocytes under physiological conditions, but upregulated when astrocytes become reactive as a result of an insult. As cellular accumulation around the injury can be the result of cell proliferation, we administered BrdU to the mice via drinking water, starting one day before the injury and continuing until the timepoint of death; we then quantified BrdU^+^ glial cells. Here, mild trauma of degree III and IV induced a significant increase in glial cell proliferation at 3 days after CHI (Fig. 2f–h), independent of the studied cell type, indicating that increased severity of the CHI exacerbates early glial reactions and especially glial proliferation. The data showing the reaction of astrocytes, specifically the GFAP upregulation and their proliferation, suggest that the increase in the number of GFAP^+^ astrocytes in the mild CHI degree I to III is mainly the result of already existing astrocytes that start expressing GFAP after CHI rather than an increase in the proliferation of reactive astrocytes (Fig. 2b,e,h, Supplementary Fig. S2b). In contrast, in the injury of degree IV the increase in number of reactive (GFAP^+^) astrocytes results from both, upregulation of GFAP and increased proliferation (Fig. 2e,h, Supplementary Fig. S2b). As we could find a robust reaction of all glial cell types in injuries of severity degree IV, we optimized our setup to reproducibly induce CHI of degree IV (80.23% of all cases), with other outcomes occurring in under 20% of the cases, to better study the reaction of glial cells (Fig. 1b). From now on, we concentrated our study on injuries with a severity degree IV.

### The glial cell reaction after CHI is spatially and temporally specific

**Figure 3:**
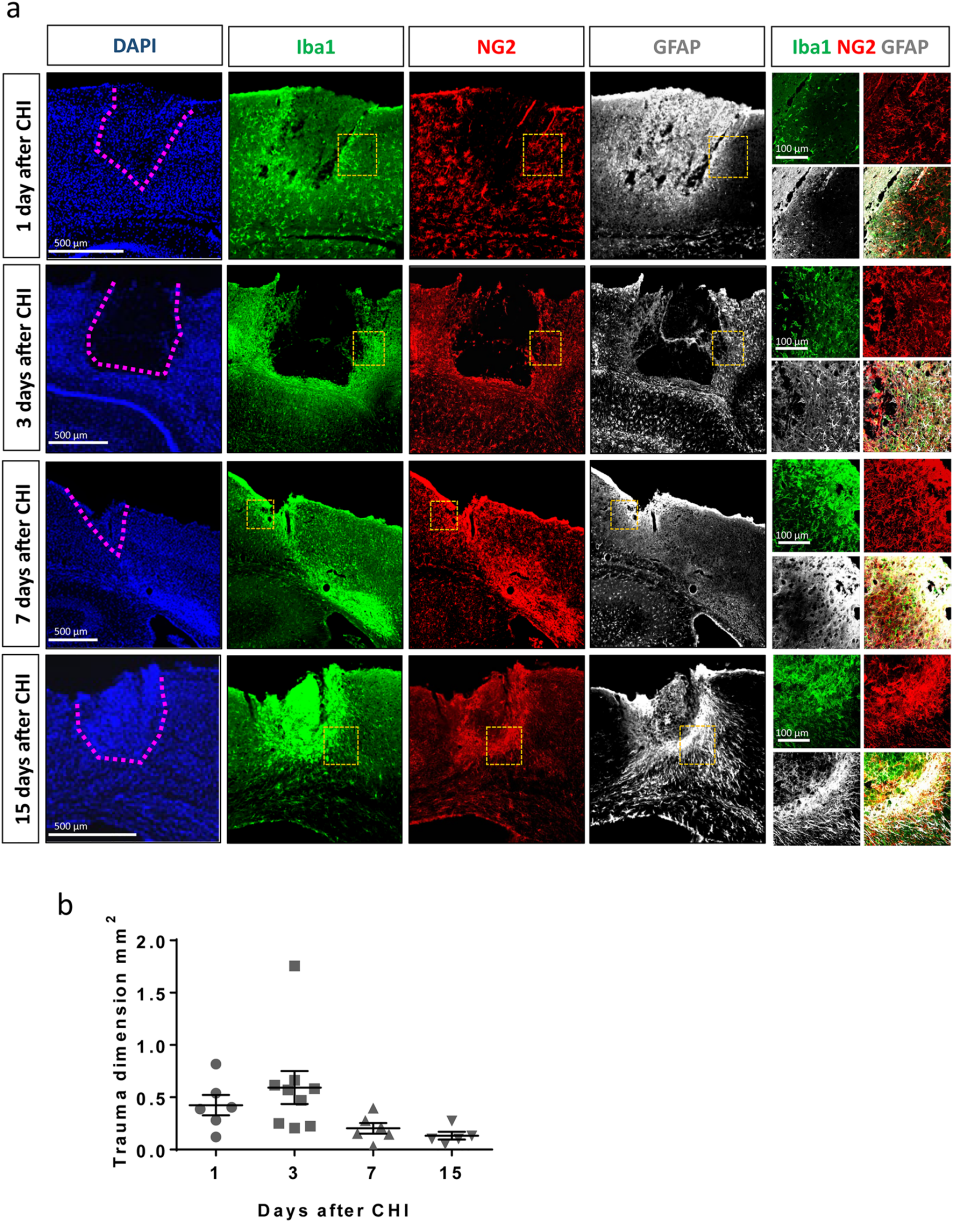
Spatial reaction of glial cells after CHI and reduction of lesion size over time. (**a**) IHC shows the progressive accumulation of Iba1^+^ cells around and within the trauma site (the borders of the trauma site are highlighted by the pink dotted line in the left panel), while NG2-glia and GFAP^+^ astrocytes surround the lesion. Yellow dotted squares show the position of the higher-magnification images (right panels), used to highlight the colocalization of the different markers. (**b**) No significant reduction in the primary trauma dimension per slice is observed over time (one-way ANOVA with Tukey's post hoc test). Data are presented as the mean ± SEM.

The acute reaction of glial cells to CHI is necessary to minimize tissue damage, re-establish tissue homeostasis and limit tissue loss. To better understand this complex process, we monitored the reaction of the different glial cell populations 1 day (d), 3 d, 7 d, and 15 d after trauma in mice with higher severity (with skull fracture and bleeding formation; Fig. 3a). The primary trauma site is clearly visible one day after CHI as an area devoid of cellular Iba1- and NG2- staining (black area in Supplementary Fig. S1). At this timepoint, strong, diffuse, nonspecific background staining for GFAP could be observed; however, this does not reflect GFAP expression within astrocytes or other cells. Specific astrocytic upregulation of GFAP, which indicates reactive astrocytes, was not visible in the area surrounding the trauma site until 3 days after CHI. The accumulation of microglia and NG2-glia at the border of the trauma site starts between 1 and 3 days after CHI and becomes increasingly clear at later timepoints. Seven days after CHI, a layered arrangement of glial cells can be observed: NG2-glia and Iba1^+^ cells invade the trauma tissue, while GFAP^+^ astrocytes are mainly present at the trauma borders. High expression of Iba1 and NG2, but not GFAP, is also visible alongside the white matter tracts of the corpus callosum as a smear that is directed laterally from the midline. When we studied the breakdown of the blood brain barrier by IgG staining, we could similarly detect increased blood content at the 7 days timepoint laterally to the injury core, although we cannot exclude prior blood infiltration that may have taken place between 3 and 7 days after trauma (Supplementary Fig. S3). Interestingly, this blood infiltration seemed to be completely resolved one week later. At the same timepoint, the core area of the TBI shows intense Iba1- staining, enclosed by NG2^+^ glia and GFAP^+^ astrocytes that have completed the formation of a glial scar. In this study, we stereotactically generated the CHI, making the location of the induced primary injury, which is purely mechanical, reproducible. However, the injury develops differently from animal to animal, leading to a distinct formation of secondary damage, including the destructive and self-propagating biological changes in cells and tissues that lead to their dysfunction or death over hours to weeks after in the initial, primary injury. The distinct and differential development of the secondary trauma also explains the differences in localization of the images in relation to the bregma. However, for our analysis, we only used slices with a by eye visible trauma core. During the initial acute phase after trauma, the injury core, an area lacking glial cells, is clearly visible and widely varies in dimension, while a slight but not significant reduction in the trauma core dimension is progressively observed (Fig. 3b).

### The reaction of the different glial populations to CHI is temporally controlled

Having detected that different glial populations display unique dynamics after trauma, we investigated the glial reaction to CHI in more detail. In particular, we followed the cellular changes in Iba1^+^ cells, NG2-glia, and GFAP^+^ astrocytes in terms of cell numbers and proliferation. For the latter, we used the thymidine analog BrdU that intercalates into the DNA of dividing cells as a marker of overall cell proliferation after the trauma, and we used Ki67 to label actively proliferating cells at different timepoints after injury.

**Figure 4:**
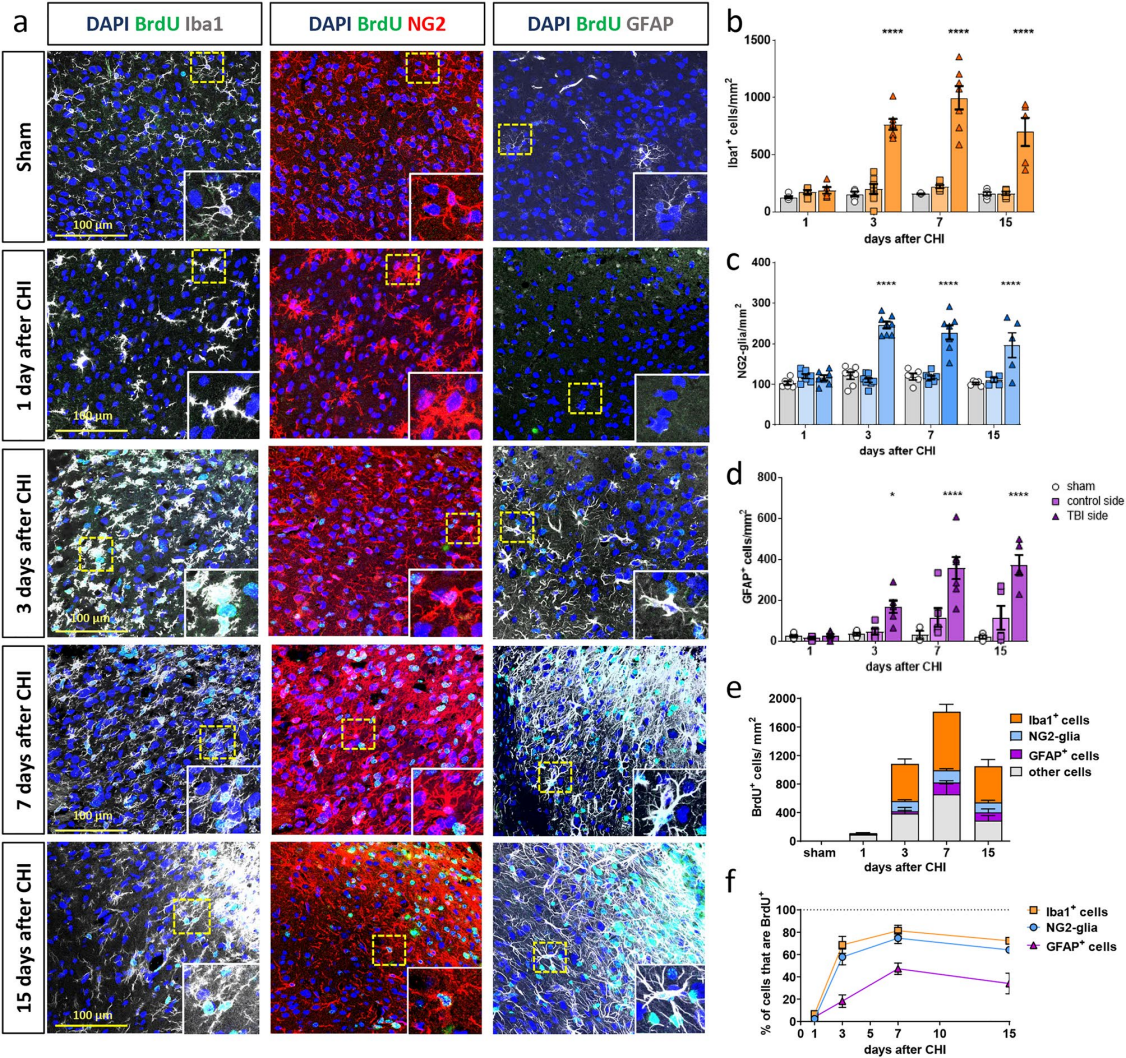
Glial cell-specific reaction to CHI over time. (**a**) IHC analysis of Iba1^+^ cells (in gray, left panel), NG2-glia (in red, middle panel), GFAP^+^ astrocytes (in gray, right panel), and proliferating BrdU^+^ cells (in green) shows morphological changes at different time points after CHI. In the images, CHI is located in the upper right corner. Higher magnification images of single cells (yellow dotted line) are shown in insets. (**b**) Quantification of Iba1^+^ cell-, (**c**) NG2-glia-, and (**d**) GFAP^+^ astrocyte-numbers that accumulate around the TBI at different timepoints after CHI. (**e**) Quantification and identity of BrdU^+^ cells, representing all cells that have proliferated between the timepoint of injury and the timepoint of sacrifice; (**f**) percentage of the total Iba1^+^ cells, NG2-glia, and GFAP^+^ cells that are also BrdU^+^ at the different time points. Statistical analysis: two-way ANOVA with Tukey’s post hoc test for multiple comparisons. *p < 0.05, ****p < 0.0001. Data are presented as the mean ± SEM.

**Figure 5:**
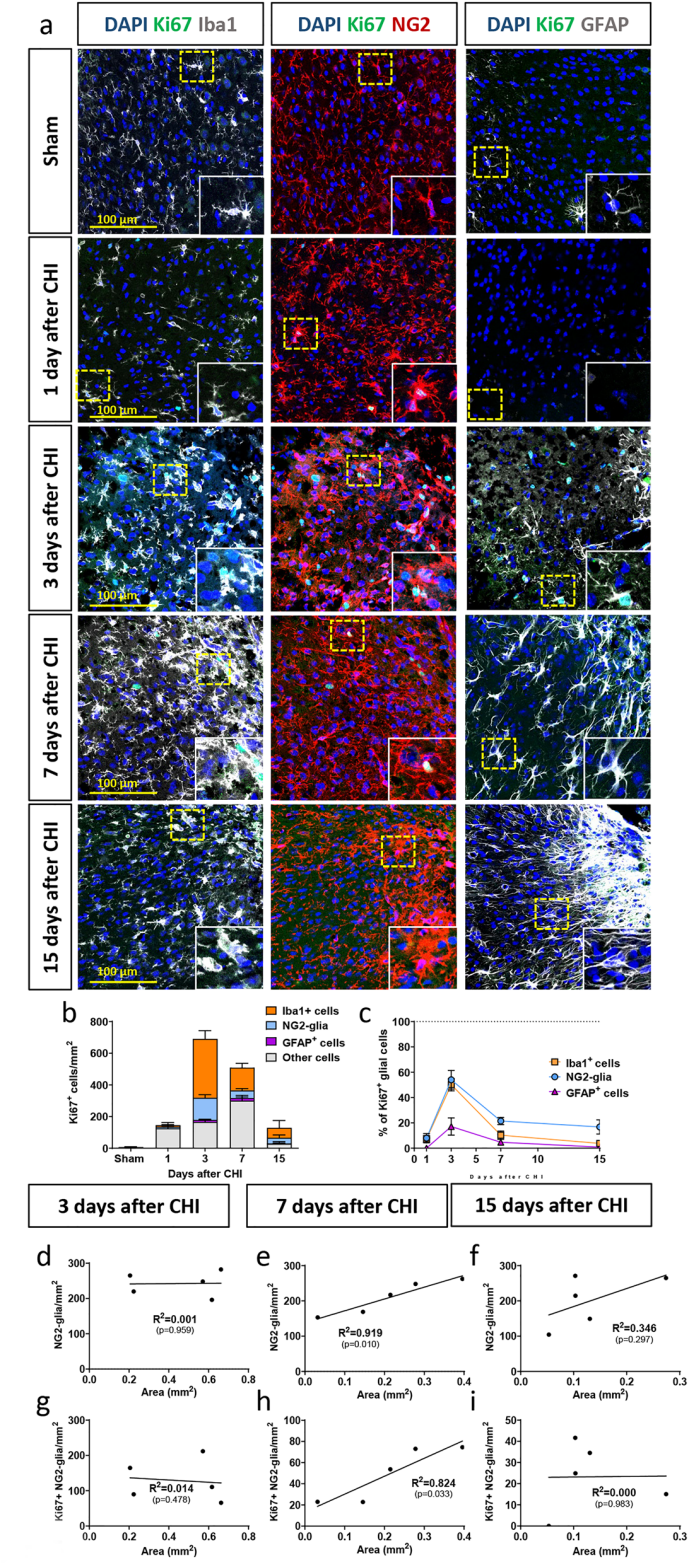
Active proliferation of glial cells changes after injury over time. (**a**) IHC analysis of Iba1^+^ cells (in gray, left panel), NG2-glia (in red, middle panel), GFAP^+^ astrocytes (in gray, right panel), and actively proliferating Ki67^+^ cells (in green) after CHI. In the images, CHI is located in the upper right corner. Higher magnification images of single cells (yellow dotted line) are shown in insets. (**b**) Quantification and identity of Ki67^+^ cells, which represent the acute proliferating cells at the timepoint of sacrifice; (**c**) percentages of all Iba1^+^ cells, NG2-glia, and GFAP^+^ cells that are also Ki67^+^ at the different time points. Correlation of lesion size at 3 (**d**,**g**), 7 (**e**,**h**) and 15 (**f**,**i**) days after trauma with the total number of NG2-glia (**d**–**f**) and Ki67^+^ NG2-glia (**g**–**i**). A correlation between the size of the lesion and the reaction of NG2-glia could only be observed at 7 days after trauma (**e**,**h**). Data are plotted along with the line of fit determined by linear regression analysis. Significance was calculated with R^2^ and p < 0.05.

Iba1 is expressed by brain-resident microglia and infiltrating macrophages, which enter the brain parenchyma through the disturbed blood brain barrier (BBB). Microglia after injury show different morphological states, with ramification, enlargement of processes, and even an amoeboid shape (Fig. 4a and inlays). At this stage, microglia are visually indiscernible from macrophages, and the two cell types work together for optimal removal of cell debris. Iba1^+^ cells showed a sevenfold increase in their abundance around the trauma site between 3 and 15 days after CHI (Fig. 4b). This is paired with intense proliferation, which we were able to track through Ki67 and BrdU staining. We observed that active proliferation (Ki67^+^ signal) of Iba1^+^ cells peaked at 3 days after the trauma and gradually declined but nonetheless persisted in low levels at 15 days after TBI (Fig. 5a). Accordingly, as BrdU labels all cells that have proliferated within the studied time, BrdU^+^ Iba1^+^ cells were more numerous, with their numbers peaking at 7 days after TBI, indicating intense BrdU incorporation into the DNA due to proliferation in the preceding days (Fig. 4e,f, Supplementary Fig. S4). Indeed, more than 80% of all Iba1^+^ cells were proliferating within the acute phase of the CHI response (Fig. 4e,f), and this reaction was not limited to the ipsilateral side but even extended to the contralateral side (Supplementary Fig. S5).

The reaction of NG2-glia, studied by using the marker NG2, starts early after injury with a clearly visible change in morphology that is temporally restricted to the first day after CHI. At this time point, NG2-glia are hypertrophic but not yet proliferative. As with Iba1^+^ cells, active proliferation for the vast majority of NG2-glia peaked at 3 days after CHI but sharply decreased at later timepoints (Fig. 4a,c,e–f and Fig. 5a–c).

Astrocytes start to become reactive -as shown by GFAP upregulation- at 3 days after CHI and extend their processes to form the astrocytic scar that is visible at 7–15 days after trauma (Fig. 4a,d). These astrocytes show not only morphological changes but also reactivity (GFAP^+^) after CHI. The highest percentage of BrdU^+^ GFAP^+^ cells can be observed at 7 days after CHI, but as the population of actively proliferating astrocytes (Ki67^+^ GFAP^+^ cells) is low at this timepoint (Fig. 5a–c), we conclude that the peak of active proliferation of GFAP^+^ astrocytes takes place between 3 and 7 days after CHI, as has also been described for other injury models^12^ (Figs. 4e,f, 5b,c, Supplementary Fig. S4).

### The reaction of NG2-glia depends on injury size

After observing that each glial cell type has a spatially and temporally defined reaction, we sought to identify different injury-related parameters that could predict cellular reaction after CHI. We chose to analyze lesion size, as it can be easily measured and compared across different animal injury models. Therefore, we quantified the total numbers of Ki67^+^ cells and concentrated on NG2-glia numbers and their proliferation properties. We excluded day 1 after injury from this analysis because glial cells do not show any major proliferative reaction at this early timepoint (Figs. 4e,f, 5b,c). At three days after injury, when the reaction of NG2-glia and Iba1^+^ cells peaks while the reaction of astrocytes is only starting, we could not observe a correlation between trauma size and the number or proliferation of glial cells. Interestingly, when we analyzed the 7 days timepoint, we could indeed find a correlation of the injury size to the total number of NG2-glia as well as to the number of actively proliferating NG2-glia (Ki67^+ ^NG2^+^). Notably, this correlation was resolved at 15 days post injury.

## Discussion

TBI is a major cause of mortality and disability worldwide and imposes a great burden on patients, as survivors are likely to develop neurological and cognitive disturbances that require long-term specialized care. Despite intensive research efforts, treatment options for the consequences of neurotrauma remain limited, as preclinical findings often fail to translate to clinical settings^1,8^. In this study, we performed a comprehensive characterization of the acute glial reaction to neurotrauma in a mouse model of CHI that might better fill the actual gap between preclinical findings and a clinical setting.

While several models of TBI exist, FPI, CCI, and SWI have been the most widely used. These models have been praised for their easy standardization and reproducibility of results, which could correlate to TBI outcomes in patients. However, SWIs account for a minor portion of TBIs in patients^23^, and similar to the CCI and FPI models, the SWI model relies on a complex surgical procedure—a craniotomy—which alone can cause intracerebral bleeding, edema formation, inflammation, and worsening of neurological scores^24^. Furthermore, in the cases of CCI and FPI, the direct impact on the brain causes extensive tissue loss and limits the predictive validity of the models^7,9^. As a craniotomy itself can lead to glial reaction^22^ and can provide confounding results, we decided to use a CHI model and optimize it to obtain a rather strong trauma that would induce consistent glial cell reaction.

The weight-drop model is generally used to mimic mild TBI, which is more frequently observed in patients. This procedure is less invasive than open-skull procedures because it requires only sedation and skin incision to expose the skull, which is directly hit by the dropped weight^20,21^. Interestingly, mice that underwent such an injury do not show any strong abnormalities when monitored by the neurological severity score (NSS) or by their motor behavior^21^. One frequent criticism of the CHI model is its limited reproducibility^9^. Indeed, after CHI, we reported different degrees of severity, and mice were divided into categories regarding the appearance of only brain concussion (degree I), only hairline skull fracture (degree II), only intracerebral bleeding formation (degree III), or a combination of skull fracture and bleeding (degree IV), reflecting the possible TBI outcomes also occurring in patients.

In our study, we focused on understanding the acute cellular reaction of glial cells to neurotrauma to unveil cellular processes that can be targeted or supported for therapeutic purposes after TBI. Surprisingly, we did not observe a substantial difference in the glial cell reaction between the group with only concussion (degree I), hairline skull fracture (degree II), and sham group. Although there are indications that skull fracture alone worsens the neurological severity score in mice^25^, our data show an acute glial cell reaction only upon increased severity of the injury (degrees III and IV), where intracerebral bleeding is also present. Our findings could be supported by the clinical observation that hematoma formation in patients after TBI was paired with increased oxidative damage, vasogenic edema and cytotoxic edema, as well as heme toxicity^26^. For these reasons, hematoma in patients is considered as an adverse prognostic factor, leading to progressive neurological deterioration and increased morbidity and mortality after TBI^27,28^. Intracerebral bleeding could indeed be one but not the only explanation for our findings since its presence in the different injury outcomes was accompanied by a strong glial cell reaction. Indeed, as already known from animal models with intracerebral hemorrhage, the extravasation of blood into the brain provokes brain damage in multiple ways: structural tissue damage is directly induced and cytotoxicity as well as inflammation are introduced by invading peripheral lymphocytes, macrophages, and monocytes as well as by blood-derived components such as immunoglobulins, heme and its degradation products, and damage-associated molecular patterns (DAMPs)^29^. All these changes after injury could also directly affect the activation of the different resident glial cells in the brain. However, which factors are specifically needed for this activation is still partially unresolved.

Microglia for example, are known to be activated by hemorrhagic DAMPs, e.g., nucleic acids, proteins, lipid mediators, and other molecules released from necrotic and damaged tissue. Within minutes, these cells react through morphological changes, cytokine secretion, and reactive oxygen and nitrogen species production, as shown with CCI, focal cerebral ischemia (FCI), and weight-drop models^30,31^. In our model, Iba1^+^ cells showed changes only in the stronger severity injury model, where skull break and bleeding was present already at early timepoints after CHI. The observed progressive change from activated to amoeboid shape is supposedly associated with increased phagocytic and inflammatory activity^30^. When microglia appear amoeboid, they are visually indistinguishable from macrophages, which are also able to express Iba1, albeit at lower levels. For this reason, both cell types are probably included in our quantifications, therefore we refer to them as Iba1^+^ cells. However, other TBI models revealed that the extravasation of macrophages in the brain parenchyma does not occur until approximately one week after TBI and is mainly limited to the core of the insult area^32^. Therefore, the large increase in Iba1^+^ cell numbers and their accumulation around the TBI observed at 3 days after CHI mostly involves microglia, whereas, at day 7 and 15, both cell types could be involved in the inflammatory processes.

Iba1^+^ cells increase their number through intense proliferation, which we followed through both, Ki67 and BrdU staining. Consistent with studies performed with other TBI models, we identified the most acute proliferative phase of microglia within the first 3 days after trauma^12,22,33^. As proliferation and therefore Iba1^+^ cell counts gradually decline over the following timepoints, interventions aiming to limit Iba1^+^ cell number increase should be initiated within this timeframe. It is important to mention that microglia can increase their numbers in the area surrounding the trauma by migration even from relatively distant regions^34^. The mobility of microglia after trauma has been documented in other injury models but could not be addressed within our experiment. Furthermore, we also found proliferating BrdU^+^ microglia on the contralateral side of the brain, supporting the idea that TBI causes global brain inflammation in the long term. This further highlights the importance of understanding how and where immune cells react to an injury, mainly in case one wants to address their reaction pharmacologically for therapeutic purposes.

Previous data have shown, that only invasive injuries such as SWI or cerebral ischemia, rather than noninvasive injuries such as chronic amyloidosis or neuronal cell death, would lead to a reaction of astrocytes and their dedifferentiation^18,35^. This kind of injuries are generally accompanied by intracerebral bleeding, which leads to tissue disruption and cell death. This suggests that a disturbance of the blood brain barrier is needed for a pronounced glial reaction. At 7 days after TBI, an area of intense Iba1- as well as NG2- staining is visible as a smear along the white matter tract of the corpus callosum, directed away from the midline, suggesting ongoing reactive gliosis in the subregions distant from the injury center. At the same timepoint, IgG staining revealed only moderate blood leakage, possibly suggesting that blood brain barrier restoration and wound closure had already started.

NG2-glia are the least understood among glial cells, and their reaction to TBI was long overlooked until recent findings showed that reactive NG2-glia play a role in brain trauma and wound closure^16,36^. Also in this study, we observed after CHI a rapid change in the morphology of NG2-glia, followed by increased proliferation peaking at 3 days after trauma and resulting in an increase in the total number of NG2-glia. This increase contributed to their accumulation, which was restricted to the area around the injury core. These data confirmed previous observations derived from other animal models of TBI following craniotomy^12,15,16,22^ and were recently found to facilitate wound closure^16^. The accumulation of NG2-glia around the injury core is also known to be supported by short-range migration, and we showed here that the severity as well as the size of the injury can affect the changes in NG2-glia homeostasis and their reaction.

Similar factors were previously observed to alter the NG2-glia reaction following SWI^16^. NG2-glia at the injury site might contribute to tissue damage and promote recovery through the release of NG2 protein, a chondroitin sulfate proteoglycan (CSPG4) that physiologically aggregates and forms the extracellular matrix. In fact, a tight and amorphic accumulation of extracellular NG2 protein at the injury border is clearly visible in our immunostaining results. NG2 can also be produced by microglia^37,38^, while astrocytes release other types of CSPGs^39,40^ to facilitate wound closure. When this deposition is altered, such as in NG2-knockout animals, an exacerbated astrocyte reaction, prolonged blood brain barrier disruption, and slower lesion resolution after SWI were documented^41^.

The astrocytic reaction to TBI, termed reactive astrocytosis or astrogliosis, is already shown in different TBI models^42,43^. Astrogliosis is able to inhibit scar formation after TBI, increase chronic inflammation, and enhance BBB leakiness as well as edema formation, thereby strongly affecting recovery^44^. As most astrocytes in the parenchyma of the cerebral grey matter do not express GFAP under physiological conditions but upregulate it in pathology, GFAP is a well-accepted marker protein for reactive astrocytosis indicating injury or pathology. After TBI in human, GFAP is released in the extracellular milieu and in the blood within minutes, with its amount correlating with trauma severity^45–47^. As shown in other studies, intracellular upregulation of GFAP in astrocytes of the cerebral grey matter has only started at 3 days after trauma. From this time point onward, astrocytes reorganize their cellular structure, become hypertrophic, prolong their processes (polarization) to reach the borders of the trauma site, and establish the glial scar^48^. At 7 days after trauma, the initial structure of the scar was already visible and at 15 days, scar formation was complete and impenetrable: high IgG and Iba1- staining in the trauma core indicated that brain isolation from the periphery had been restored, while NG2- and GFAP-staining highlighted the borders of the scar. Compared to other glial cells, astrocytic proliferation was delayed and peaked between 3 and 7 days after trauma, similar to what was observed after SWI^12^, while after CCI, astrocytic proliferation was already observed at day 3^22^. Their reaction could be stimulated by factors correlating with the severity of the injury. This is plausible if we consider that astrocytes are fundamental for the physiological maintenance of the BBB and its proper restoration after trauma and are thought to be a valid target for the therapy of hemorrhagic stroke^44,49,50^.

In summary, this study demonstrated that microglia, astrocytes, and NG2-glia react to TBI, each class with its unique temporal and spatial response that follows a rather standardized procedure even across different animal models and that can be dramatically altered depending on the trauma severity and the dimension of the injury. An extensive understanding of the course of neuroinflammatory events in this translational brain injury mouse model could open novel avenues for future therapeutic approaches for TBI.

## Methods

### Animals

Male NMRI mice were housed in a specific-pathogen-free animal facility at Ulm University under standardized conditions (12 h/12 h dark–light cycle; temperature between 20 and 24 °C) and with ad libitum access to food and water. All animal experiments were performed in compliance with the guidelines on the use of animals and humans in neuroscience research as revised and approved by the Regierungspräsidium Tuebingen (Tuebingen, Germany). Ethical approval is obtained from animal ethics committee of the Regierungspräsidium Tuebingen (Tuebingen, Germany).

### CHI model

Adult (12–20 weeks old) mice were provided BrdU (bromodeoxyuridine, 1 mg/mL and 1% sucrose) in the drinking water starting 1 day before CHI induction until they were sacrificed. The BrdU water was given in dark flasks and was renewed every 2–3 days to ensure freshness. As drinking water intake and therefore BrdU intake is subject to variability among mice, only mice that showed postmortem positivity to BrdU immunostaining were included in the quantifications. Furthermore, we also compared the cell proliferation of areas far away from the injury site to confirm a rather equal BrdU consumption. On the day of CHI induction, mice were anesthetized with an intraperitoneal (i.p.) injection of ketamine hydrochloride 10%, 100 mg/kg (Pfizer Pharma, Karlsruhe, Germany) and xylazine 2%, 5 mg/kg (Bayer Health Care, Germany) before undergoing experimental CHI with a standardized weight-drop device (for details see^20,21^). For the procedure, the skull was exposed through a longitudinal incision of the skin, and a focal blunt injury was achieved by dropping a 330 g metal rod with a diameter of 2 mm on the left side of the skull from a height of 2.5 cm onto an impact site at approximately the following stereotaxic coordinates: anteroposterior coordinate = − 1.2 mm and mediolateral coordinate = − 1.5 mm. The falling weight was manually stopped after the impact to avoid rebound injury. Mice were then left to recover in a warm cage, and buprenorphine analgesia (Temgesic; Essex Pharma, Munich, Germany) was administered subcutaneously (0.03 mg/kg body weight) immediately after trauma and again at 6 h and 24 h after trauma. For the whole duration of the experiment, mice were closely monitored, and only animals with no visible signs of pain were included in the study. Sham animals underwent the same experimental procedure except for the weight-drop injury.

### Postmortem sample preparation

For the analysis of the glial reaction after different severity degrees of the injury, mice were sacrificed 3 days after CHI or sham procedure (sham: n = 5, degree I: n = 4; degree II: n = 3, degree III: n = 3; degree IV: n = 7; n reflects single animals). For the analysis of glial cell reaction, mice were sacrificed 1 day, 3 days, 7 days, and 15 days following CHI (5–7 mice per group). In the latter case, only mice that showed an injury of severity degree IV were included in the study, along with sham mice (sham: n = 5–6 per timepoint (1, 3, 7 and 15 days), CHI: n = 5–7 per timepoint (1, 3, 7 and 15 days); n reflects single animals). For postmortem analysis, animals were anesthetized with an i.p. injection of a mixture of ketamine (100 mg/kg) and xylazine (10 mg/kg) diluted in a 0.9% NaCl solution. For tissue fixation, transcardial perfusion was performed, first with 25 mL of ice-cold PBS to flush out the blood, followed by 50 mL of ice-cold 4% PFA in PBS. Brains were extracted and further postfixed in 4% PFA for 2 h, transferred to a solution of 30% saccharose in PBS at 4 °C overnight for cryoprotection, and stored at 4 °C until use.

### Histology and immunofluorescence staining

For immunohistochemistry, the brain samples were cryosectioned into 20 µm-thick slices, and each slide contained 3–4 sequential brain slices. Different cellular markers were stained on each slice. Only slides that visibly included the core of the trauma were selected and included in the study. Brain slices were permeabilized and blocked in a solution of 5% BSA in PBS with 0.5% Triton and incubated with primary antibodies in blocking solution overnight at 4 °C. After washing with PBS, slices were incubated with secondary antibodies in blocking solution for 2 h at room temperature. For nuclear antibodies, heat-mediated antigen retrieval was needed and was performed with sodium citrate (10 mM, pH 6.0, 0.05% Tween 20) before incubation with the primary antibody. When both nuclear and nonnuclear antibodies were used at the same time, only nonnuclear antibodies were incubated at first and were then fixed with 4% PFA before antigen retrieval. After antigen retrieval, the slices were incubated with both nuclear and nonnuclear antibodies. The following primary antibodies were used: rabbit α-NG2 (1:250, Merck #AB5320), rat α-Ki67 (1:250, Thermo Fisher Scientific #14-5698-82), rat α-BrdU (1:250, Abcam #ab6326), guinea pig α-Iba1 (1:500, Synaptic Systems #234004), mouse α-GFAP (1:500, Sigma-Aldrich #G3893) and mouse α-S100β (1:250, Sigma-Aldrich #S2532). The following secondary antibodies were used for immunodetection of the aforementioned primary antibodies: α-rat Alexa Fluor^®^ 488 (1:500, Thermo Fisher #A21208), α-mouse IgG Alexa Fluor^®^ 647 (1:500, Dianova #115-606-072), α-guinea pig IgG Alexa Fluor^®^ 647 (1:500, Thermo Fisher #A21450), and α-rabbit Cy^®^ 3 (1:500, Dianova #711-165-152). Finally, slices were washed with PBS, incubated with DAPI at a 1:1000 dilution for nuclear counterstaining for 10 min, and then mounted on a slide with Aqua-Poly/Mount. In glial proliferation studies, the marker NG2 was always present to consistently determine the area of imaging across different slices and samples and used together with a proliferation marker (KI67 or BrdU) and with either Iba1 or GFAP. This facilitated the recognition of the same area to use for image acquisition.

### Image acquisition and cellular quantification analysis

For image acquisition, only slides containing the CHI core were considered. Overview images of the trauma area were acquired with a Keyence BZ-9000 BioRevo microscope (Keyence, Neu-Isenburg, Germany) with filters for DAPI, FITC/Alexa Fluor 488, Texas Red/Alexa Fluor 568/594 and Cy5 at 10× and 20× magnification. These images were then used for the analysis of the trauma dimension, which was manually done through Fiji (based on ImageJ 1.48i) software, considering a total of 3 slices per animal and 7–10 mice per timepoint. For cellular quantification around the area of trauma, the perilesional area adjacent to the CHI was specified (yellow area in Supplementary Fig. S1), and image acquisition was performed with a Leica SPE confocal microscope at 40× magnification and analyzed through Fiji (ImageJ). For every immunostaining, 4–12 images were quantified for each mouse, depending on the availability of the slides containing the injury core. Only cells that were positive for the selected markers and contained visible DAPI signal were taken into consideration in the analysis. The Iba1 antibody was used to identify microglia and macrophages, and the GFAP antibody was used to identify reactive astrocytes. For NG2-glia quantification, in addition to NG2 staining, the presence of a ramified cellular morphology and a localization not adjacent to any blood vessel were applied as criteria to avoid confusion with NG2^+^ pericytes. All data were analyzed in a blinded manner. All quantitative data are reported as the mean ± SEM. No statistical method was used to determine the sample size; instead, it was purely based on previous studies in the field. The significance of our quantification was tested by one-way or two-way ANOVA with Tukey’s post hoc test for multiple comparisons. Data were considered significant at p value < 0.05 = *, p value < 0.01 = **, p value < 0.001 = *** and p value < 0.0001 = ****. All statistical analyses were performed with GraphPad Prism 9.

### Ethical approval

All animal experiments were performed in accordance with the guidelines on the use of animals and humans in neuroscience research as revised and approved by the Regierungspräsidium Tuebingen. Ethical approval is obtained from animal ethics committee of the Regierungspräsidium Tuebingen (Tuebingen, Germany).

### ARRIVE guidelines

The study is reported following the recommendations of the ARRIVE guidelines.

### Supplementary Information

Supplementary Figure 1.

Supplementary Figure 2.

Supplementary Figure 3.

Supplementary Figure 4.

Supplementary Figure 5.

## Data Availability

The raw data that support the findings of this study are available from the corresponding author upon reasonable request.
