# Supplementary material for: Glial cells react to closed head injury in a distinct and spatiotemporally orchestrated manner
Source: Sci Rep. 2024 Jan 30;14:2441. doi: 10.1038/s41598-024-52337-4 (PMC10825139; doi:10.1038/s41598-024-52337-4)
Supplement: Supplementary file 1 — Supplementary Figure 1. [file 41598_2024_52337_MOESM1_ESM.pptx]

## Slide 1
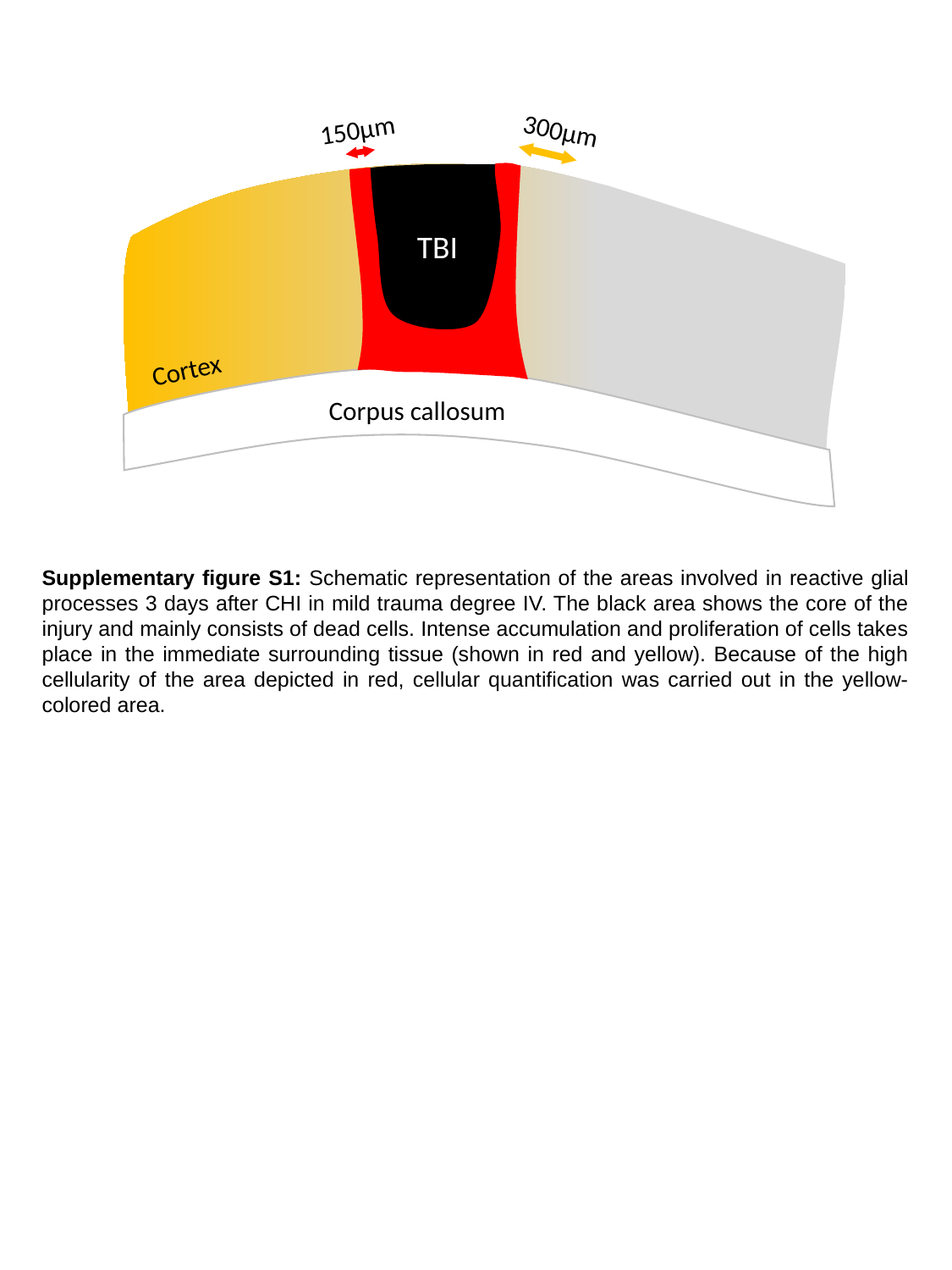

150µm
300µm
TBI
Cortex
Corpus callosum
Supplementary figure S1: Schematic representation of the areas involved in reactive glial processes 3 days after CHI in mild trauma degree IV. The black area shows the core of the injury and mainly consists of dead cells. Intense accumulation and proliferation of cells takes place in the immediate surrounding tissue (shown in red and yellow). Because of the high cellularity of the area depicted in red, cellular quantification was carried out in the yellow-colored area.
