# Supplementary material for: Glial cells react to closed head injury in a distinct and spatiotemporally orchestrated manner
Source: Sci Rep. 2024 Jan 30;14:2441. doi: 10.1038/s41598-024-52337-4 (PMC10825139; doi:10.1038/s41598-024-52337-4)
Supplement: Supplementary file 2 — Supplementary Figure 2. [file 41598_2024_52337_MOESM2_ESM.pptx]

## Slide 1
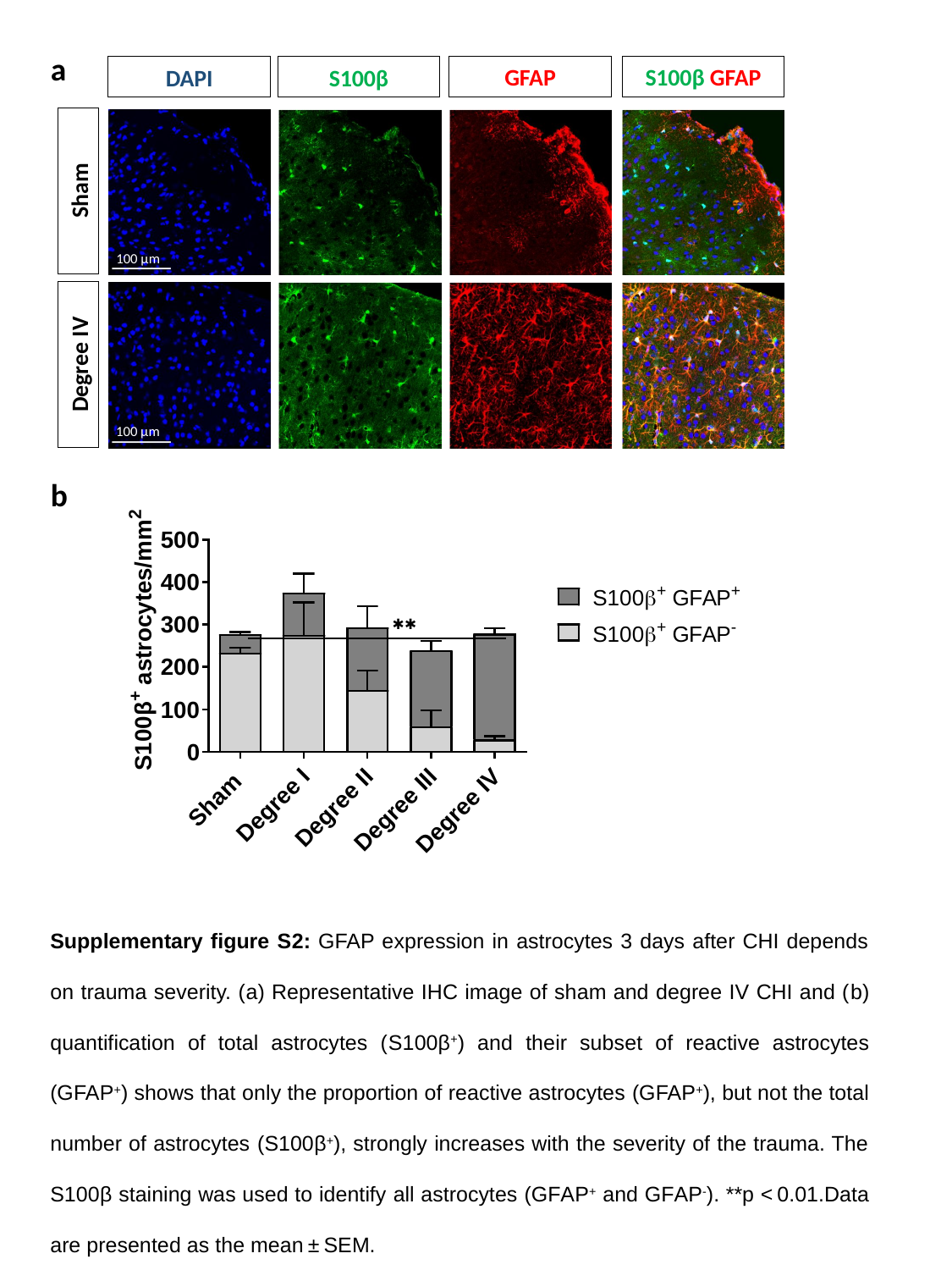

a
GFAP
S100β GFAP
DAPI
S100β
Sham
Degree IV
100 µm
100 µm
b
Supplementary figure S2: GFAP expression in astrocytes 3 days after CHI depends on trauma severity. (a) Representative IHC image of sham and degree IV CHI and (b) quantification of total astrocytes (S100β+) and their subset of reactive astrocytes (GFAP+) shows that only the proportion of reactive astrocytes (GFAP+), but not the total number of astrocytes (S100β+), strongly increases with the severity of the trauma. The S100β staining was used to identify all astrocytes (GFAP+ and GFAP-). **p < 0.01.Data are presented as the mean ± SEM.
