# Supplementary material for: Glial cells react to closed head injury in a distinct and spatiotemporally orchestrated manner
Source: Sci Rep. 2024 Jan 30;14:2441. doi: 10.1038/s41598-024-52337-4 (PMC10825139; doi:10.1038/s41598-024-52337-4)
Supplement: Supplementary file 3 — Supplementary Figure 3. [file 41598_2024_52337_MOESM3_ESM.pptx]

## Slide 1
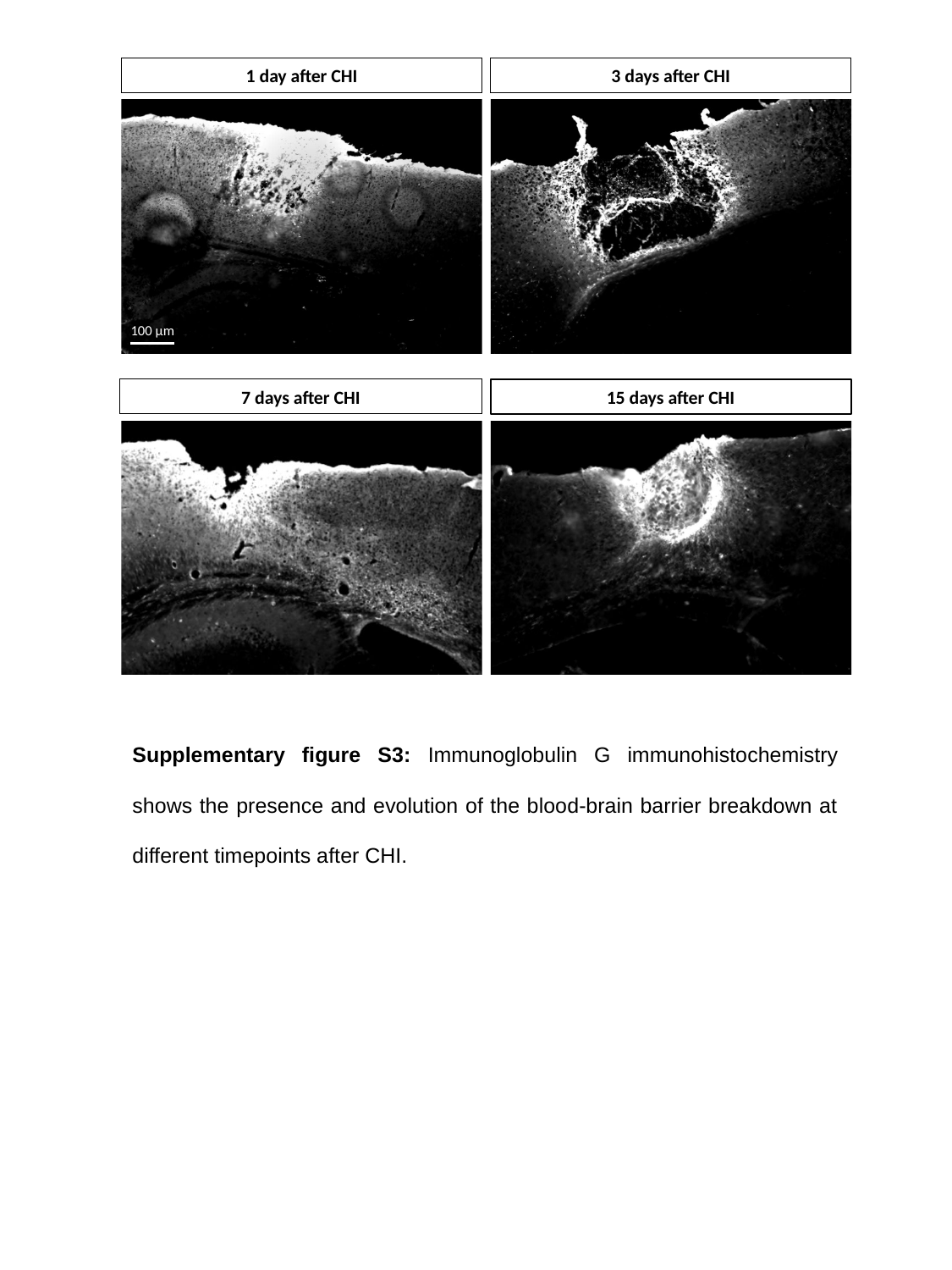

1 day after CHI
3 days after CHI
7 days after CHI
15 days after CHI
100 µm
Supplementary figure S3: Immunoglobulin G immunohistochemistry shows the presence and evolution of the blood-brain barrier breakdown at different timepoints after CHI.
