# Supplementary material for: Glial cells react to closed head injury in a distinct and spatiotemporally orchestrated manner
Source: Sci Rep. 2024 Jan 30;14:2441. doi: 10.1038/s41598-024-52337-4 (PMC10825139; doi:10.1038/s41598-024-52337-4)
Supplement: Supplementary file 5 — Supplementary Figure 5. [file 41598_2024_52337_MOESM5_ESM.pptx]

## Slide 1
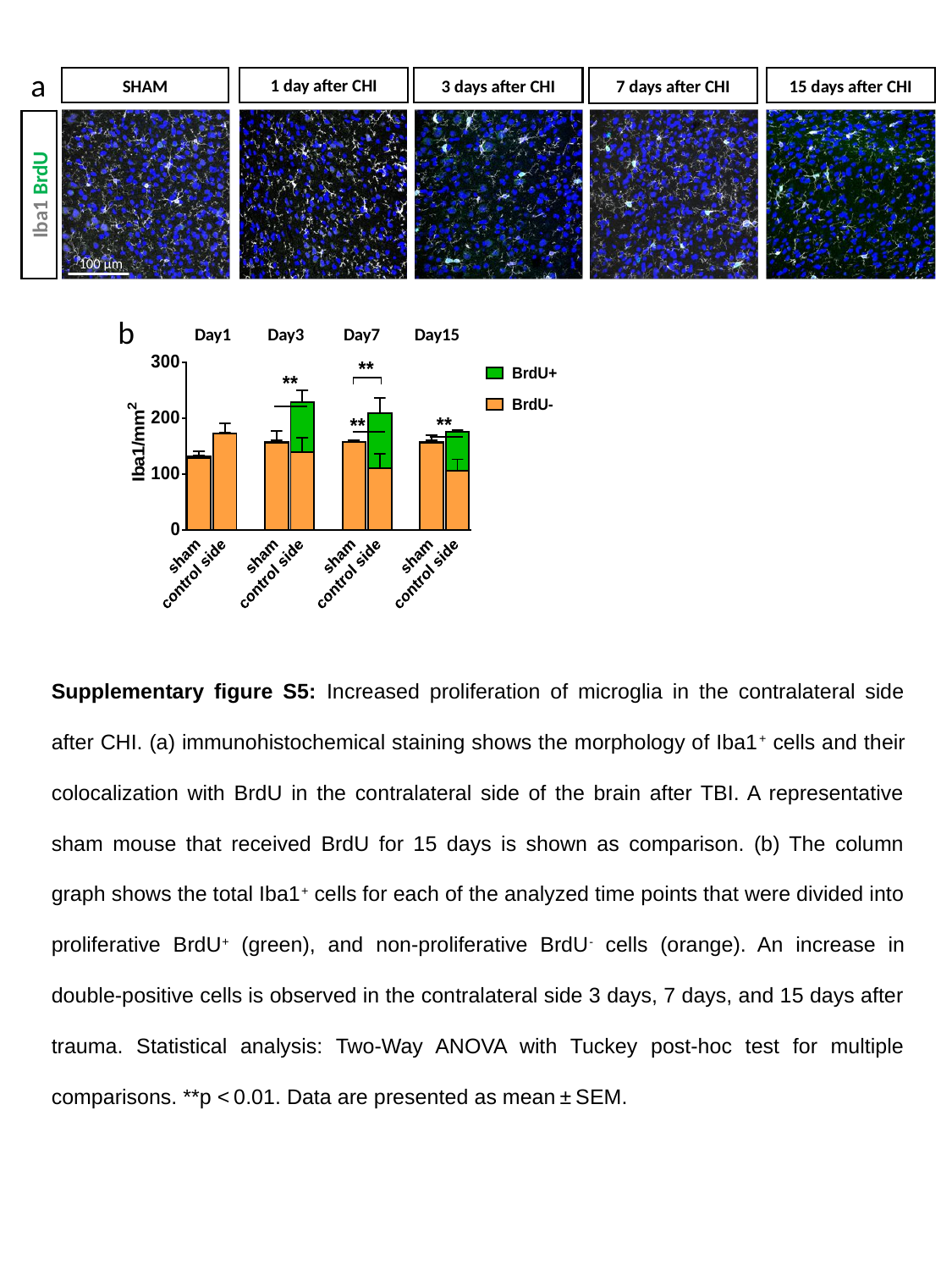

a
1 day after CHI
7 days after CHI
3 days after CHI
15 days after CHI
SHAM
Iba1 BrdU
100 µm
b
Day15
Day7
Day1
Day3
Supplementary figure S5: Increased proliferation of microglia in the contralateral side after CHI. (a) immunohistochemical staining shows the morphology of Iba1+ cells and their colocalization with BrdU in the contralateral side of the brain after TBI. A representative sham mouse that received BrdU for 15 days is shown as comparison. (b) The column graph shows the total Iba1+ cells for each of the analyzed time points that were divided into proliferative BrdU+ (green), and non-proliferative BrdU- cells (orange). An increase in double-positive cells is observed in the contralateral side 3 days, 7 days, and 15 days after trauma. Statistical analysis: Two-Way ANOVA with Tuckey post-hoc test for multiple comparisons. **p < 0.01. Data are presented as mean ± SEM.
